# The Focus of Attention in Visual Working Memory: Protection of Focused Representations and Its Individual Variation

**DOI:** 10.1371/journal.pone.0154228

**Published:** 2016-04-21

**Authors:** Anna Heuer, Anna Schubö

**Affiliations:** Experimental and Biological Psychology, Philipps-University Marburg, Marburg, Germany; University of Verona, ITALY

## Abstract

Visual working memory can be modulated according to changes in the cued task relevance of maintained items. Here, we investigated the mechanisms underlying this modulation. In particular, we studied the consequences of attentional selection for selected and unselected items, and the role of individual differences in the efficiency with which attention is deployed. To this end, performance in a visual working memory task as well as the CDA/SPCN and the N2pc, ERP components associated with visual working memory and attentional processes, were analysed. Selection during the maintenance stage was manipulated by means of two successively presented retrocues providing spatial information as to which items were most likely to be tested. Results show that attentional selection serves to robustly protect relevant representations in the focus of attention while unselected representations which may become relevant again still remain available. Individuals with larger retrocueing benefits showed higher efficiency of attentional selection, as indicated by the N2pc, and showed stronger maintenance-associated activity (CDA/SPCN). The findings add to converging evidence that focused representations are protected, and highlight the flexibility of visual working memory, in which information can be weighted according its relevance.

## Introduction

Attention and working memory have for a long time been studied as distinct domains. In recent years, however, systematic investigations of their interrelations have revealed substantial overlap [1–5] with bidirectional influences [3,6]. In the present experiment, the attentional modulation of maintenance in visual working memory (VWM) is investigated. VWM is the system that allows us to maintain and manipulate visual information, thereby enabling us to interact with our immediate environment. Although, at each moment, we are flooded with visual information, our ability to retain this information is highly limited (e.g., [7,8]). As our environment and our goals are constantly changing, information permanently gains or loses relevance. A mechanism for dynamically modulating the information that is maintained in VWM according to changes in relevance would thus be highly advantageous to make efficient use of this limited system. Recent studies have demonstrated that attention can be oriented towards memory representations, allowing for a flexible modulation of VWM content (e.g., [9–13]). Attentional orienting in the mnemonic and the perceptual domain have been shown to be highly similar with regard to behavioural benefits (and costs) and neural networks involved [10,12,14–20]. In order to selectively manipulate attentional orienting during the maintenance stage of VWM, these studies used retrocues, which were presented during a retention interval to orient attention to items previously presented at specific locations.

The mechanism underlying the benefit observed for memory items cued in this way is not yet entirely understood. What are the consequences of focusing a subset of items for this subset and for those items that are no longer focused? Previous studies attempting to answer this question can broadly be assigned to one of two theoretical accounts. They either concluded that attention serves to reduce memory load, or that attention is employed to protect focused items. Previous findings shall in the following be presented within this framework. Before that we briefly introduce some key terms as we use them here.

### Focused, defocused and refocused information in VWM

The present experiment is based on the idea that there is an internal focus of attention (FoA) that implies different states of representations in VWM: within and outside this FoA (see [21–23]). There is a growing body of evidence supporting this notion [24–28]. Focused items are those items which are currently maintained and in the FoA within VWM. Defocused items have recently been focused, but have, at least intermittently, left the FoA. These items can be refocused, that is, brought back into the FoA. This terminology is based on the terminology used by Rerko and Oberauer [29], but differs with respect to the meaning of “unfocused” information. Rerko and Oberauer [29] defined “unfocused” information as “information that has not been focused (attended) after initial encoding.” Our different understanding results from a different concept of the FoA. There is an ongoing debate on whether the FoA is a single-item focus (e.g., [22]) or whether it can hold more than one item. The present experiment is based on the idea of a flexible FoA, as suggested by Cowan et al. [30], according to which attention can “zoom in” on single items or “zoom out” to grasp multiple items. According to Cowan’s and our understanding, several items may be initially focused, and the FoA would only then zoom in on specific items when information with respect to differences in the relevance of the maintained items becomes available, that is, after a retrocue has singled out one or several items. Following this line of reasoning, all memorized items are in the FoA after encoding, and accordingly there is no information in VWM that has never been focused after initial encoding.

### Protection of focused items

One scenario for the consequences of attentional selection within VWM is that attention is employed to protect focused items, while defocused items are subject to faster decay. There are two major predictions which may be derived from this “protection account”. First, memory for continuously focused items should be better than for unfocused or intermittently defocused items. Second, defocused items should remain available for recall and comparison with a probe, that is, a test stimulus. Previous studies provide some support for both predictions.

Matsukura, Luck, and Vecera [31] provided early evidence that attention is used to protect cued items from degradation, rather than prioritizing these items at recall. Murray, Nobre, Clark, Cravo, and Stokes [32] were able to show that retrocues increased the likelihood of recall, and that more items were available after retrocueing than in a no-cue condition. The authors propose that attention does not only protect items from decay, but that it can even serve to restore items which would otherwise be unavailable to retrieval mechanisms. A number of studies have shown that retrocueing renders an item more robust against interference from subsequent stimuli [33–36]. As pointed out by Pertzov, Bays, Joseph, and Husain [37], protection from degradation and enhancing robustness against interference are by no means mutually exclusive. Retrocueing may protect selected items from interference, which may either arise from novel incoming stimuli or from other items residing in memory.

As to the second prediction derived from the protection account, there is evidence that unfocused information remains available and that defocused items remain strengthened [29] with recently focused items being more easily recollected when only little time has elapsed from focusing them [38]. Poorer memory for defocused information might be due to forgetting as a function of time [29] or due to enhanced forgetting as compared to when no subset of items is focused [37], but for the purpose of the present experiment it suffices to assume that defocused items remain available, yet memory for them is worse than for continuously focused items.

### Memory load reduction

Kuo et al. [11] suggested that attention may be employed to reduce memory load. They asked participants to perform a VWM task in which a retrocue was either informative or neutral. The Contralateral Delay Activity (CDA, see [39], also called Sustained Posterior Contralateral Negativity (SPCN), see [40], or Contralateral Negative Slow Wave (CNSW), see [41], and in the following referred to as CDA/SPCN), a lateralized event-related potential (ERP) component that is sensitive to the number of maintained items [39,42], revealed a larger amplitude attenuation after an informative retrocue than after a neutral one. Thus, retrocues that reduced the number of relevant items also reduced delay activity associated with the maintenance of information. The authors interpreted this finding as attentional selection in terms of a reduction of the number of items being maintained. But they did not test performance for those items rendered irrelevant by the retrocue to make sure that they were no longer being maintained.

That uncued items were never tested in the study of Kuo et al. might have been a crucial factor in determining the fate of these items. Indeed, it has been demonstrated that representations are excluded from VWM following so-called “directed-forgetting cues”, which are almost always valid [43]. In this extreme situation, when information is either relevant or absolutely irrelevant, is seems highly beneficial for an efficient use of the limited VWM system to discard any uncued items. These findings do not, however, allow for a conclusion as to whether or not the defocusing of representations necessarily leads to an exclusion of these representations. Given that a selection of relevant information already occurs for encoding into VWM [3], the situation created by valid retrocues that some of this information is absolutely irrelevant shortly thereafter during maintenance is rather unlikely to be frequently encountered outside the laboratory. A situation that might better reflect everyday life would be that some information is currently more relevant, warranting attentional protection, while other information might still be important in the future and thus worth holding on to.

### Rationale of the experiment

In the present experiment, we addressed two issues. First we examined the fate of intermittently defocused items. Do they remain available in a scenario in which they may become task-relevant again? And if they do, is there a cost associated with defocusing, or does refocusing boost these items to a level similar to that of continuously focused items? Second, we investigated whether higher individual efficiency of attentional orienting within VWM was associated with a greater retrocueing benefit. In a double-retrocue paradigm, a first retrocue directed attention to two memory items. Thereafter, the FoA was either constant throughout the entire trial (Hold condition), or it had to be expanded to again include either one (Add 1 condition) or two (Add 2 condition) previously defocused items. A neutral retrocue condition provided the baseline for when no subset of representations was focused. Participants were to judge whether the probe item was one the items indicated by the second retrocue. Behavioural performance and two ERP components associated with either the efficiency of attentional selection (N2pc) or VWM maintenance (CDA/SPCN) were analysed. Both the N2pc and the CDA/SPCN are lateralized components which appear as enhanced negativity at posterior electrode sites contralateral to a respective visual hemifield.

The N2pc is usually observed about 200–300 ms after stimulus onset (e.g., [44,45]) and has been shown to be sensitive to the number of targets selected or individuated [46–50]. N2pc amplitude increased with an increasing number of targets, reaching an asymptote at three to four items. Importantly, N2pc asymptote has been shown to differ as a function of participants’ behavioural efficiency [50] or tracking capacity [46]. Low performers exhibited weaker amplitude modulations with increasing target numbers, reaching an asymptote at a smaller number.

The CDA/SPCN appears approximately 275 ms after stimulus onset and typically persists throughout the retention interval, although the amplitude tends to decline over time, presumably due to an increase in ipsilateral activity [42]. The prevailing notion is that the CDA/SPCN reflects the number of items being maintained in VWM, as its amplitude is sensitive to the number of memory representations, reaches a limit when the mean capacity limit is exceeded, and reflects individual differences in VWM capacity [39]. There is, however, still some debate on what exactly it is the CDA/SPCN reflects. For example, CDA/SPCN amplitude appears to be sensitive to stimulus characteristics other than number, such as stimulus identity [51] or precision of representations [52]. It has also been used as an index of tracking load in multiple object tracking tasks, in which the CDA/SPCN was interpreted as an index of the number of attended items [46,53].

Behavioural performance and the CDA/SPCN were analysed to shed light on whether defocused items are still available for refocusing and recall. In our task, there was a relatively high likelihood (67%) that at least one initially uncued item would become relevant again, and different implications of this situation for intermittently defocused items were conceivable. First, if attentional selection in VWM was mainly employed to reduce memory load, these items might be excluded. Even with the refocusing likelihood of the present experiment, uncued items would remain irrelevant in a substantial number of trials, and excluding these items would relieve VWM by reducing memory load from four to two items. Behavioural performance for these items should then be close to chance level, and CDA/SPCN amplitudes in the cued conditions should be reduced after the first retrocue, as compared to the neutral retrocue condition. Second, in line with the protection account, intermittently defocused items might still be available for refocusing and recall. Overall, this should show in performance well above chance level, and a differential refocusing and weighting of items after the second retrocue is likely to reflect in diverging CDA/SPCN amplitudes in the cued conditions compared to after the first retrocue. A more detailed comparison of the continuously focused and intermittently defocused items in the two Add conditions will further elucidate the consequences of this refocusing: Upon refocusing, initially uncued items might either be “preserved”at their current strength, or they might be “boosted“. Accordingly, performance for intermittently defocused items might be either worse than or equivalent to performance for continuously focused items. Moreover, the comparison of performance for the continuously focused items in the two Add conditions with performance for the continuously focused items in the Hold condition will provide insight into whether the inclusion of additional items in the FoA affects the maintenance of items already in the FoA.

To establish whether the individual efficiency of attentional deployment is related to the magnitude of the retrocueing benefit, correlations between the N2pc modulations following each retrocue and the behavioural retrocueing benefit were computed. Stronger N2pc modulations compared to the neutral condition, indicating higher attentional efficiency, were expected to be associated with larger retrocueing benefits.

Additionally, on the group level, analysis of the N2pc served to ensure that both retrocues induced differential attentional processing. As N2pc amplitude has been shown to scale with target number, N2pc amplitudes after the first retrocue were expected to be larger in the cued conditions than in the neutral condition, in which no attentional selection should take place. A further divergence of N2pc amplitudes in the cued conditions after the second compared to after the first retrocue was expected, with larger amplitudes in the Add conditions than in the Hold condition.

## Methods

### Ethics statement

The experiment was approved by the Ethics Committee of the Faculty of Psychology at Philipps-University Marburg, and conducted according to the principles of the Declaration of Helsinki. All participants provided informed written consent prior to the experiment.

### Participants

Twenty-six students of Philipps-University Marburg participated in the experiment. Nine participants showed too many trials (> 40%) containing EEG artifacts. Analyses were performed on the remaining participants (11 female, 6 male, mean age = 22 years). All participants were naive to the purpose of the experiment, and had normal or corrected-to-normal visual acuity and normal colour vision. Visual acuity was tested with a Landolt C eye chart and colour vision with Ishihara’s tests for colour deficiency [54].

### Apparatus and stimuli

Participants were seated in a comfortable chair in a dimly lit and electrically shielded room, facing a monitor placed at a distance of approximately 104 cm from their eyes. They were instructed to maintain fixation during the experimental trials.

Stimuli were presented on a 22” screen (1680 x 1050 px) using E-Prime 2.0 software (Psychology Software Tools, Inc.). All stimuli were presented against a grey background. The colour of each memory item was randomly selected from a set of nine colours (blue, green, orange, pink, purple, red, turquoise, white and yellow). A given colour could appear no more than twice in an array, and only once in each hemifield. The colour of the test probe item was either randomly selected from the set of colours of the squares which were marked as relevant by the second retrocue (match trials) or from the set of colours not present in the attended hemifield on the respective trial (nonmatch trials). Each coloured square subtended 1.32° of visual angle in size. There were eight fixed positions for the eight squares of the memory array (four in each hemifield, two in each quadrant), which were arranged on an imaginary circle with a radius of approximately 4.95° of visual angle. Memory items within each hemifield were positioned at distances of 3.58° of visual angle from centre to centre. The memory items to the left and right of the vertical midline were positioned at a distance of 4.95° of visual angle from the centre of a square in one hemifield to the centre of the square on the other side of the vertical midline. The probe item was presented centrally to prevent any anticipatory external attentional orienting to other locations on the display. The precue subtended 1.10° of visual angle and the fixation dot 0.17° of visual angle. Retrocues were octagrams composed of two overlapping squares (1.10° of visual angle), with each corner pointing towards one of the locations of the memory items. For cueing specific items, the respective corners were blackened.

### Procedure

**Fig 1:**
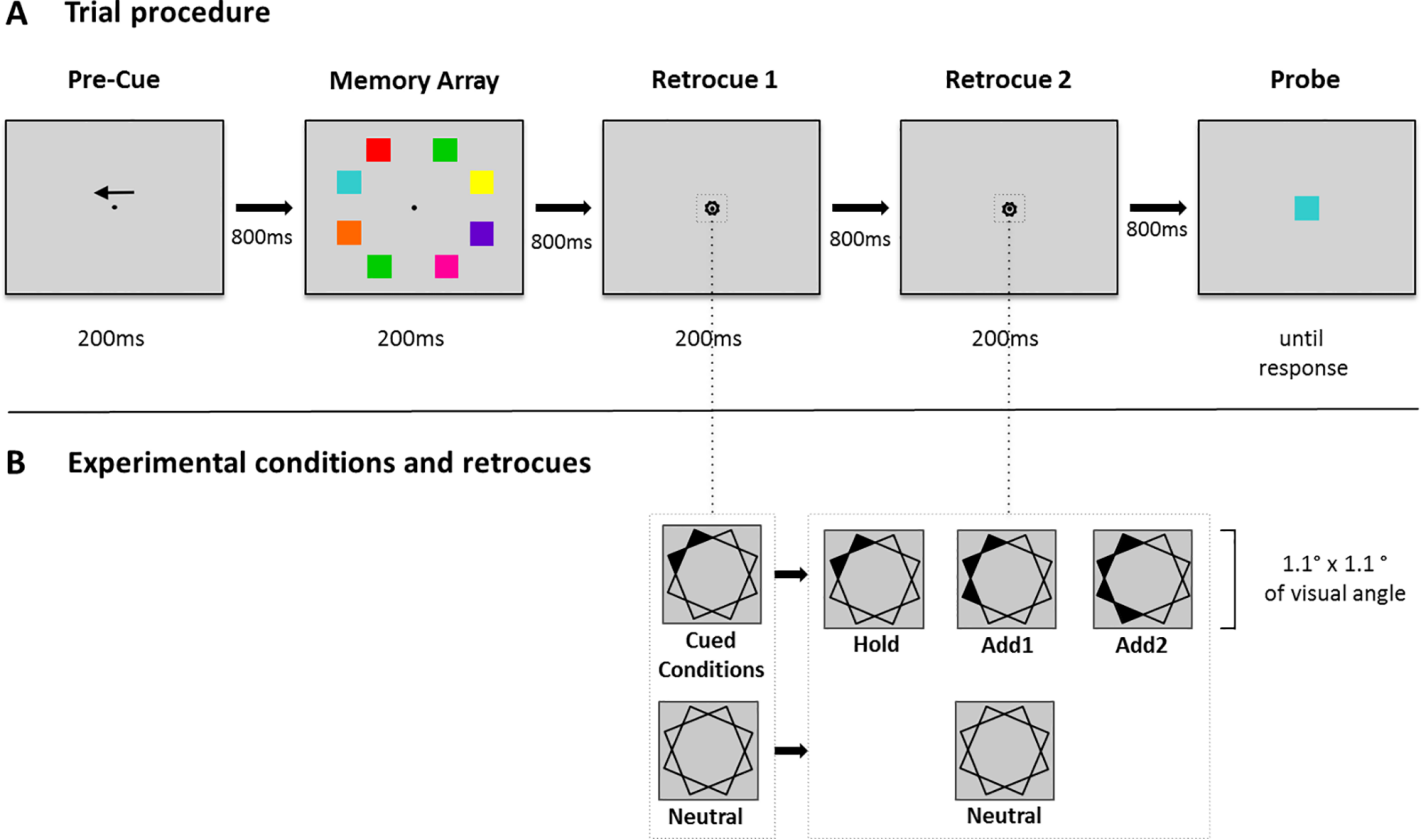
Trial procedure, experimental conditions and retrocues. A trial started with an arrow presented for 200 ms above fixation, indicating the relevant hemifield for that trial. After an inter-stimulus-interval of 800 ms, the memory array consisting of eight coloured squares was presented for 200 ms. Participants were instructed to memorize the squares in the respective hemifield. After a retention interval of 800 ms, a first retrocue was presented for 200 ms. In the cued conditions, this retrocue indicated two positions of previously presented memory items, i.e. the upper or lower quadrant of the respective hemifield. In the neutral condition, the retrocue provided no spatial information. After another interval of 800 ms, a second retrocue was presented for 200 ms. In the cued conditions, this retrocue was either identical to the first one (Hold), additionally pointed to the adjacent square (Add1), or marked the whole hemifield (Add2). In the neutral condition, the second retrocue was identical to the first one. After 800 ms, a central probe item was presented. Participants had to decide whether the probe was one of the items indicated by the second retrocue. Grey dotted squares show examples of the retrocues in the different experimental conditions.

The task is illustrated in Fig 1. A trial started with the presentation of an arrow presented just above the fixation dot for 200 ms, which pointed to one hemifield of the display. After an inter-stimulus interval of 800 ms, a memory array was presented for 200 ms, which consisted of four coloured squares in each hemifield. Participants had to memorize the squares in the previously indicated hemifield. After an interval of 800 ms, the first retrocue appeared for 200 ms. In the cued conditions, the first retrocue always marked either the upper or lower quadrant of the relevant hemifield. In the neutral condition the retrocue did not provide any spatial information as to the task-relevance of specific items. After another interval of 800 ms, the second retrocue was presented for 200 ms. In the cued conditions, the second retrocue was either identical to the first retrocue (Hold condition), additionally pointed to the adjacent square (Add1 condition), or marked the whole hemifield (Add2 condition). In the neutral condition, the second retrocue was identical to the first. Following the second retrocue and after an inter-stimulus interval of 800 ms, a test probe item was presented at the centre of the display until participants responded. Participants were to decide whether the probe item was the same as one of the items indicated by the second retrocue, responding match or nonmatch with a button press using their left or right index finger. The buttons were on the backside of a gamepad (Microsoft SideWinder USB) and the response assignment was balanced across participants. Accuracy was stressed, but in case of uncertainty participants were asked to decide spontaneously. The interval between trials varied randomly between 500 and 1000 ms.

There were 896 trials in total, randomized across 28 blocks of 32 trials each, with 224 trials per condition. This design introduced the following probability structure in the cued conditions. When the first retrocue was not neutral but marked two memory items, the likelihood that the second retrocue would additionally indicate at least one initially uncued item was 67%. Seeing as only the adjacent item was cued in the Add1 condition, the likelihood for that item to become relevant again was 67%, and the likelihood for the remaining fourth item only cued in the Add2 condition to become relevant again was 33%.

Testing took place on two consecutive days. The data of the first day’s session were considered practice and not entered into the analyses. The EEG was recorded on the second day. Between blocks as well as in the middle of each block, participants were given the opportunity of a short rest. After the experiment, participants filled in a questionnaire to assess strategies and other factors that might affect performance.

### Behavioural analyses

Trials with excessively long reaction times (> 2.5 SD from mean RT calculated separately for each participant) were excluded from further analysis (Hold: 2.8%, Add1: 2.7%, Add2: 2.3%, Neutral: 3.6%; on average, 2.9% of all trials). Accuracy in percent and mean reaction time (only correct responses) were analysed.

### EEG recording and analysis

The EEG was recorded continuously using BrainAmp amplifiers (Brain Products, Munich, Germany) from 64 Ag/AgCl electrodes (actiCAP) positioned according to the international modified 10–20 system. Vertical (vEOG) and horizontal electrooculogram (hEOG) were recorded as the voltage difference between electrodes positioned above and below, and to the left and right of the eyes, respectively. All channels were referenced to FCz and re-referenced offline to the average of all electrodes. Electrode impedances were kept below 5 kΩ. The sampling rate was 1000 Hz with a high cutoff filter of 250 Hz (half-amplitude cutoff, 30dB/oct) and a low cutoff filter of 0.016 Hz (half-amplitude cutoff, 6dB/oct).

The EEG was segmented into epochs of 3200 ms, starting 200 ms prior to the onset of the memory array and ending at the onset of the test probe display. The time period from -200 ms to 0 was used for baseline correction. Trials with response errors were excluded from analysis. Trials with blinks (vEOG > 80 μv; on average, 6% of all trials) and eye movements (hEOG > 50 μv; on average, 10% of all trials) were excluded from all channels. Additionally, trials in channels with other artefacts were excluded if the amplitude exceeded ± 80 μv. To assess residual eye movements towards the cued hemifield, the hEOG was quantified for each cued hemifield, condition and time window of analysis. The maximum deflection was 4.02 μv, which can be considered equivalent to an eye movement of about 0.25° [55]. In order to isolate the N2pc and the CDA/SPCN components, left (PO3, PO7) and right (PO4, PO8) parieto-occipital electrodes were averaged for each visual hemifield and experimental condition. Difference waves were then calculated by subtracting activity ipsilateral from activity contralateral to the respective hemifield.

## Results

### Behavioural results

**Fig 2:**
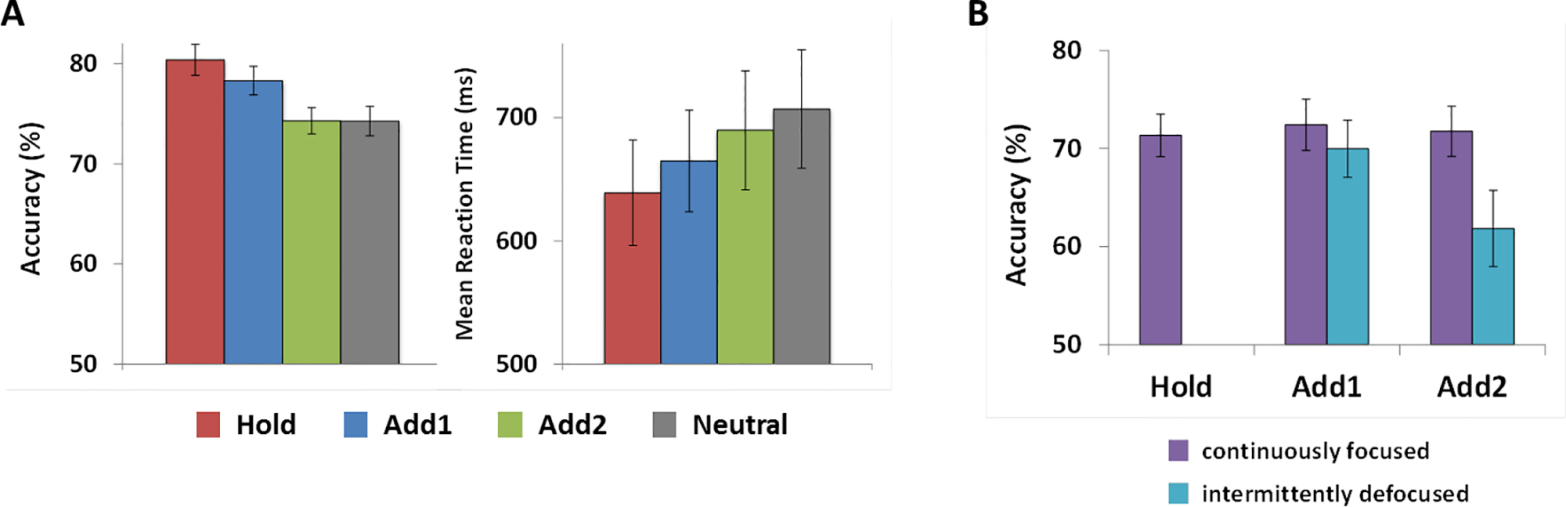
Behavioural results. **A** The left panel shows accuracy in percent and the right panel mean reaction time in ms for each experimental condition (Hold in red, Add1 in blue, Add2 in green and Neutral in grey). **B** Accuracy in percent for the different types of match probes in the Add conditions and in the Hold condition (continuously focused match probes in violet, intermittently defocused match probes in turquoise). Error bars show the standard errors of the means.

Behavioural results are plotted in Fig 2. Separate one-way ANOVAs were computed for accuracy and reaction time. Performance differed across conditions, with respect to both accuracy [F_(3,48)_ = 14.95, p < .001, partial ƞ^2^ = .48] and reaction time [F_(3,48)_ = 19.07, p < .001, partial ƞ^2^ = .54]: Participants performed best in the Hold condition [*accuracy* 80.38 ± 1.6; *RT* 639.01 ± 42.74], followed by Add1 [*accuracy* 78.31 ± 1.4; *RT* 664.86 ± 41.17], Add2 [*accuracy* 74.29 ± 1.3; *RT* 689.74 ± 48.25], and the neutral condition [*accuracy* 74.28 ± 1.5; *RT* 706.98 ± 48.02] (see Fig 2A). ANOVA contrasts revealed significant differences between Hold and Neutral [*accuracy* F_(1,16)_ = 23.59, p < .001, partial ƞ^2^ = .60; *RT* F_(1,16)_ = 65.55, p < .001, partial ƞ^2^ = .80] and Add1 and Neutral [*accuracy* F_(1,16)_ = 14.28, p = .002, partial ƞ^2^ = .47; *RT* F_(1,16)_ = 18.01, p = .001, partial ƞ^2^ = .53], and for reaction time also between Add2 and Neutral [F_(1,16)_ = 5.08, p = .039, partial ƞ^2^ = .24].

To rule out that the effects were driven by an external shift of attention induced by the retrocues, a behavioural control experiment was conducted. In this control experiment, the retrocues had no lateralized components. The results replicated the benefits observed in the present experiment: Performance was best in the Hold condition (81.18 ± 1.9), followed by Add1 (78.97 ± 1.6), the Add2 (75.13 ± 1.2) and the neutral condition (74.61 ± 1.8) (see S4 Fig for the experimental conditions, retrocues and results of this control experiment). These findings indicate that the retrocueing benefits observed in the present experiment were not driven by the lateralized aspects of the retrocues, but were the result of the voluntary focusing of attention onto representations maintained in VWM.

In a second step, results in the Add1 and Add2 condition were further analysed in order to separate accuracy performance for continuously focused and intermittently defocused match probe items (see Fig 2B). First, a two-way ANOVA with the factors condition (Add1 vs. Add2) and probe item type (continuously focused vs. intermittently defocused) was computed. Second, t-tests served to compare the intermittently defocused match probe items in each Add condition against the chance level of 50%. Third, t-tests comparing the continuously focused match probes in each Add condition against the match probes in the Hold condition were computed. This was done to test whether performance for continuously focused items in the Add conditions was equivalent to performance for the continuously focused items in the Hold condition, even though the Add condition required the additional focusing of one or two more items. The ANOVA revealed a main effect of probe item type [F_(1,16)_ = 5.96, p = .027, partial ƞ^2^ = .27] with lower accuracy for intermittently defocused items, and an interaction between the factors of condition and probe type [F_(1,16)_ = 4.93, p = .048, partial ƞ^2^ = .22], attributable to a larger difference between accuracy for continuously focused and intermittently defocused items in the Add2 condition compared to the Add1 condition. Even though accuracy for intermittently defocused items was lower than accuracy for continuously focused items, it was well above chance level in both the Add1 [t_(16)_ = 6.85, p < .001] and the Add2 condition [t_(16)_ = 3.04, p = .008]. Performance for the continuously focused items in the Add1 [t_(16)_ = 0.54, p = .595] and in the Add2 condition [t_(16)_ = 0.32, p = .76] was equivalent to performance for the match probes in the Hold condition.

### Electrophysiological results

**Fig 3:**
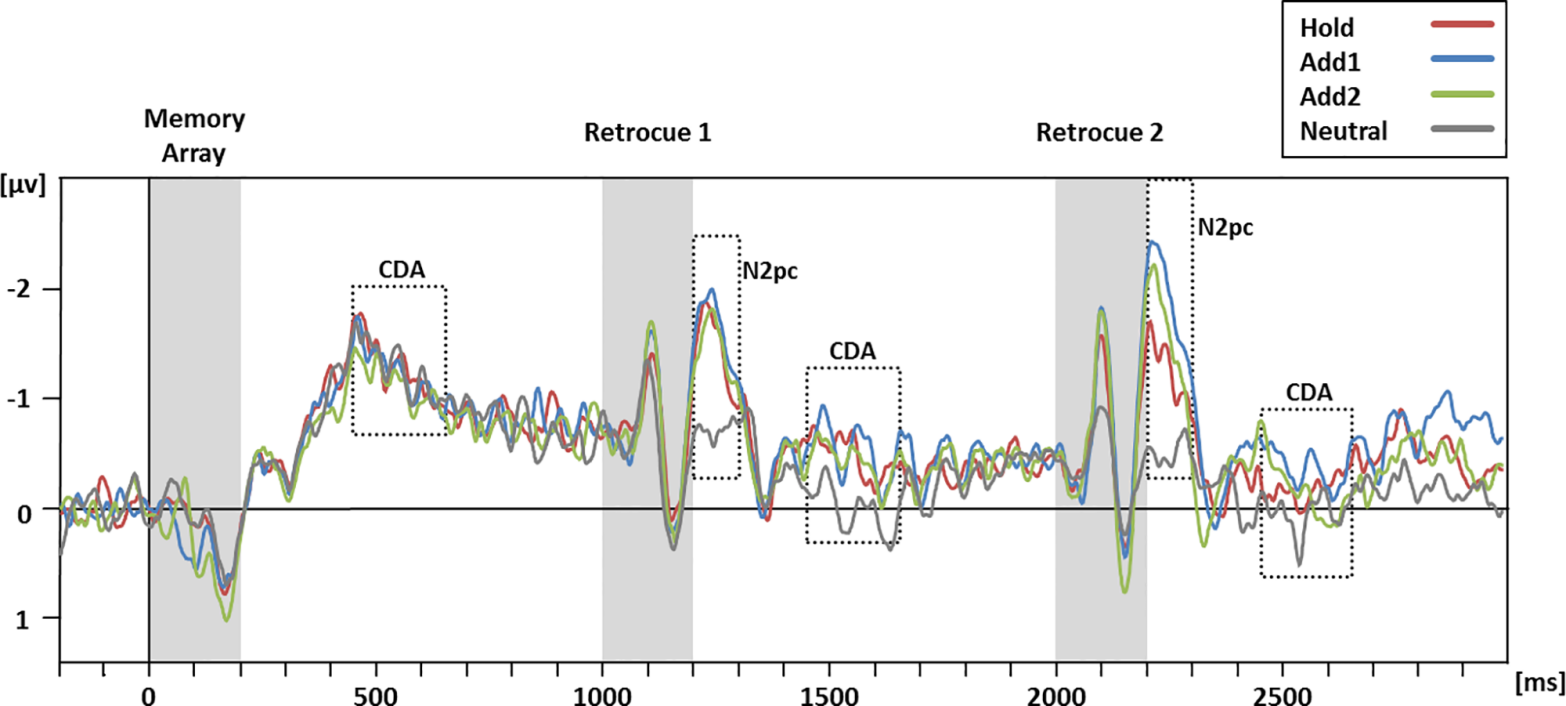
Grand-averaged ERP difference waves (contralateral activity minus ipsilateral activity). Difference waves are shown for the experimental conditions (Hold in red, Add1 in blue, Add2 in green and Neutral in grey) time-locked to the onset of the memory array averaged across parieto-occipital electrodes (PO3/PO4, PO7/PO8). Time windows of stimulus presentations are shaded in grey, the time windows for N2pc and CDA/SPCN analyses are indicated by grey dotted squares. For illustration purposes the waveforms were lowpass filtered (half-amplitude cutoff at 35 Hz, 24 dB/oct).

ERP results are plotted in Fig 3. For N2pc analyses, mean amplitudes were computed for time intervals from 200 to 300 ms after the onset of each retrocue. For CDA/SPCN analyses, mean amplitudes were computed for three time intervals, namely from 450 to 650 ms after the onset of the memory array and after each retrocue. This time window was chosen based on the interval following the memory array, for which a CDA/SPCN but no differential effects between conditions were expected. To determine the presence of the lateralized components N2pc and CDA/SPCN, two-way ANOVAs were run on the mean amplitudes for these time windows, with the factors condition (Hold, Add1, Add2, and Neutral) and laterality (ipsilateral and contralateral). For the interval following the first retrocue, an additional t-test was computed to compare the cued conditions (Hold, Add1, and Add2) directly against the neutral condition. For the cued conditions, additional three-way repeated measures ANOVAs with the factors of retrocue (first and second), condition (Hold, Add1, and Add2) and laterality (ipsilateral and contralateral) were run for both N2pc and CDA/SPCN time windows of analysis.

An N2pc was present both after the first [F_(1,16)_ = 20.75, p < .001, partial ƞ^2^ = .57,] and second retrocue [F_(1,16)_ = 24.10, p < .001, partial ƞ^2^ = .60]. Interactions between condition and laterality after both the first [F_(3,48)_ = 7.41, p = .002, partial ƞ^2^ = .23, ε = .62] and the second retrocue [F_(3,48)_ = 11.15, p < .001, partial ƞ^2^ = .41, ε = .78] revealed differences in lateralized activity and the following more specific analyses provided more insight into these differences between conditions: After the first retrocue, the N2pc observed in the cued conditions was larger than the N2pc in the neutral condition [t_(16)_ = 3.29, p = .005]. Following the second retrocue, mean N2pc amplitudes in the cued conditions diverged compared to the time window following the first retrocue. This was revealed by a three-way interaction of the factors retrocue, condition and laterality [F_(2,32)_ = 4.32, p = .022, partial ƞ^2^ = .21]. N2pc amplitudes after the second retrocue were larger in the Add1 than in the Hold condition [t_(16)_ = 3.77, p = .001]. N2pc amplitudes in the Add2 and the Hold conditions [t_(16)_ = 1.58, p = .067], and in the Add1 and Add2 conditions [t_(16)_ = 1.51, p = .075] did not differ significantly.

After presentation of the memory array, a CDA/SPCN was observed [F_(1,16)_ = 87.98, p < .001, partial ƞ^2^ = .85], which was equivalent in size for all conditions [F_(3,48)_ = 0.64, p = .590, partial ƞ^2^ = .04]. There was a significant overall CDA/SPCN [F_(1,16)_ = 7.46, p = .015, partial ƞ^2^ = .32] and an interaction between condition and laterality [F_(3,48)_ = 4.12, p = .011, partial ƞ^2^ = .21] only after the first retrocue. Similar to the N2pc results, the CDA/SPCN after the first retrocue was larger in the cued conditions than in the neutral condition [t_(16)_ = 3.12, p = .007]. There was no divergence of CDA/SPCN amplitudes in the cued conditions after the second retrocue compared to the time window following the first retrocue.

To ensure that the observed patterns in N2pc and CDA/SPCN modulations were not driven by effects in early sensory potentials attributable to different processing of the cues, all analyses for the time windows after each retrocue were rerun with the retrocue presentation time as baseline (i.e., 1000 ms to 1200 ms for the first retrocue, and 2000 ms to 2200 ms for the second retrocue). The observed patterns and all reported effects remained.

Scalp distributions of N2pc and CDA/SPCN were compared for the time window following the first retrocue. This interval was chosen because ERPs could be collapsed across the cued conditions, leading to more reliable measures. Difference waves for parietal, parieto-occipital and occipital electrode pairs were calculated, and the respective activity was normalized following the procedure described by [56]. A two-way ANOVA with the factors of time window (1200–1300, 1450–1650) and electrode position (seven posterior electrode pairs) revealed an interaction [F_(6,96)_ = 6.16, p < .001, partial ƞ^2^ = .28, ɛ = .56], demonstrating distinct scalp distributions. The N2pc showed a more ventral, and the CDA/SPCN a more dorsal distribution (see S3 Fig), which is in line with previous findings [42,46].

### Correlations between behavioural and electrophysiological measures

**Fig 4:**
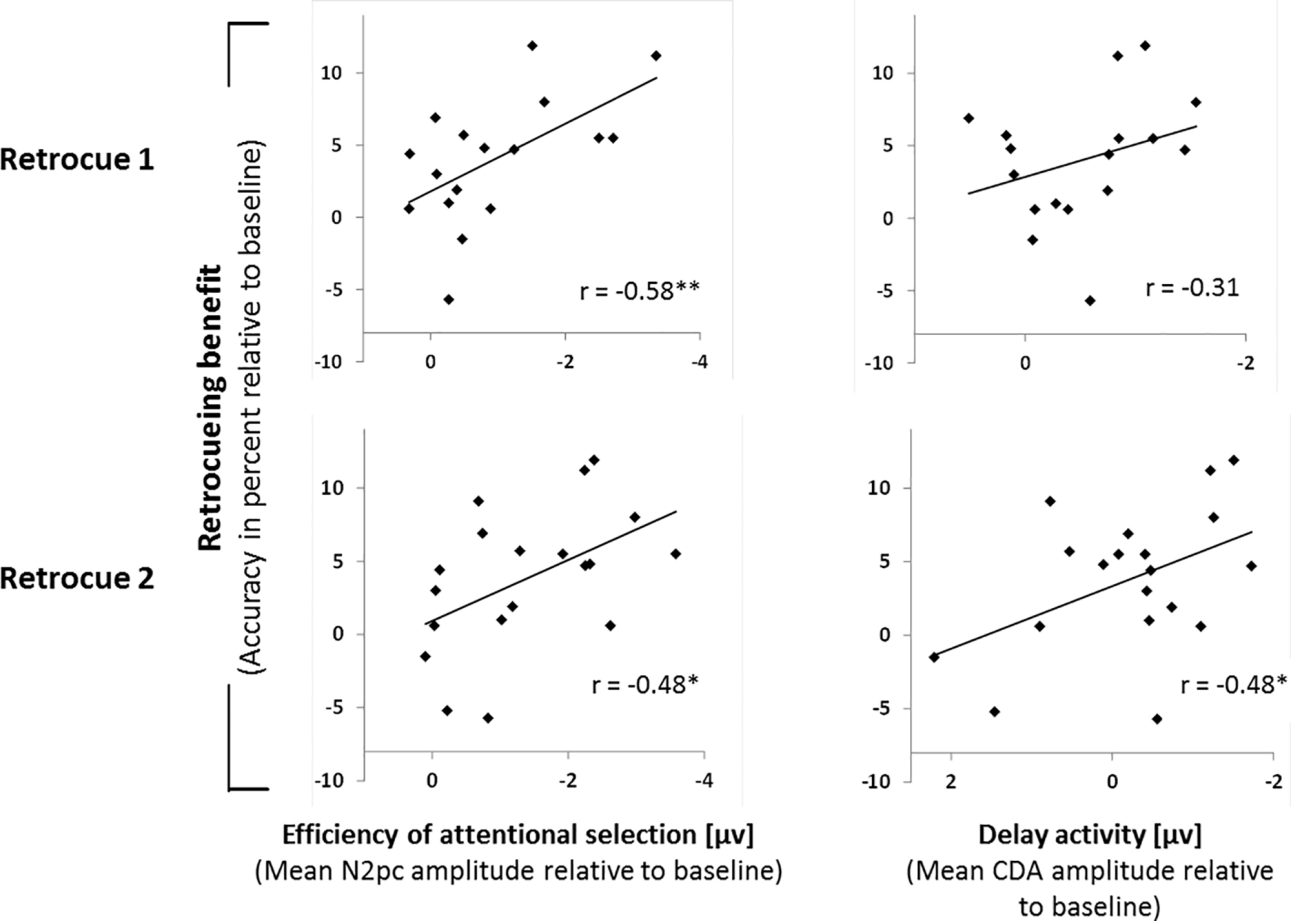
Correlations between behavioural and ERP results. The first column shows correlations between N2pc amplitude and accuracy results, and the second column correlations between CDA/SPCN amplitudes and accuracy results. For correlations in the top row, ERP measures were calculated from the time windows following the first retrocue, for correlations in the bottom row, ERP measures were calculated from the time windows following the second retrocue. All measures were computed for the Add1 condition and relative to baseline, i.e. the value of each measure in the neutral condition was subtracted from the value of the respective measure in the Add1 condition.

Scatter plots are shown in Fig 4. One-tailed correlations (Pearson’s correlation coefficient, *r*) were computed between individual N2pc amplitudes and accuracy, and between CDA/SPCN amplitudes and accuracy. This was done for the Add1 condition relative to baseline by subtracting each participant’s value in the neutral condition from the respective value in the Add1 condition. The Add1 condition was chosen, because it required attentional selection after each retrocue (unlike the Hold condition) and included a number of items that is well within mean VWM capacity (unlike the Add2 condition). Split-half reliabilities (Pearson’s correlation coefficient, *r*; one-tailed) were computed from even-numbered and odd-numbered trials and corrected for the full number of trials using the Spearman-Brown formula [57,58] (reported are corrected correlation coefficients and the uncorrected p-values). These were significant for accuracy (r = .73, p = .007), for the N2pc after both the first (r = .67, p = .020) and the second retrocue (r = .71, p = .010), and for the CDA/SPCN after the second (r = .74, p = .006), but not after the first retrocue (r = .32, p = .232).

Significant correlations were found between the retrocueing benefit and N2pc amplitude after both the first (*r* = -.576, p = .008) and the second retrocue (*r* = -.477, p = .027) (Fig 4, first column). The correlation between CDA/SPCN amplitude and retrocueing benefit was significant for the time window following the second retrocue (*r* = -.483, p = .025), but not for the time window following the first retrocue (r = -.307, p = .115) (Fig 4, second column). According to these correlations, the larger the individual efficiency of attentional selection (as indicated by N2pc differences) and the stronger the maintenance-associated activity, the larger the individual retrocueing benefit.

## Discussion

With the present experiment, we addressed two questions. First, we asked whether defocused items remain available in a scenario in which these items may become task-relevant again. The results showed that this was the case: defocused items could later be refocused and successfully recalled. Thus, the defocusing of items does not necessarily lead to their loss. Performance for these intermittently defocused items was, however, worse than for continuously focused items, indicating that a cost is associated with defocusing, and that refocusing an item cannot boost it back to the initial level. This cost appeared to be higher when the likelihood to become task-relevant was lower: Performance for intermittently defocused items was worse in the Add2 condition than in the Add1 condition (t_(16)_ = 2.85, p = .006; see Fig 2B). Whereas the likelihood to become task-relevant again was 67% for the intermittently defocused item in the Add1 condition (this item was always adjacent to the items cued by the first retrocue, and would be required to be refocused in both the Add1 and Add2 conditions), it was only 50%, on average, for the intermittently defocused items in the Add2 condition (67% for the item adjacent to the items cued by the first retrocue, and 33% for the nonadjacent item).

Similar conclusions as to the importance of task context and retrocue reliability in determining the fate of defocused items have been reached by recent studies. Zokaei et al. [59] found that an item that remains behaviourally relevant can be maintained outside the FoA and refocused, but that an item rendered very unlikely to be probed is lost from VWM. Similarly, Gunseli et al. [60] observed costs of invalid retrocueing only when the retrocue had a high reliability (80%), but not when it had a low reliability (50%). Thus, irrelevant items can be removed, or maintained items can be strategically weighted with respect to their relevance and according to a given probability structure. In the same vein, van Moorselaar et al. [61] suggested that representations in VWM can adopt different states depending on their deemed relevance, and that these states can be flexibly adjusted to changing task demands. Accordingly, items can be maintained inside as well as outside the FoA, with different degrees of robustness or vulnerability, respectively. Taken together with findings demonstrating that it is principally possible to exclude representations from memory following an almost always valid cue presented during the maintenance interval [43], the present results add support to the notion of a high flexibility of visual working memory when it comes to making the most out of its limited resources given a specific situation.

Importantly, the inclusion of intermittently defocused items in the FoA in the two Add conditions did not affect maintenance of the continuously focused items. Focused representations seem to be protected from degradation, and this protection appears to be unaffected by an expansion of the FoA and the inclusion of additional representations. Thus, the particularly robust maintenance inside the FoA provides a reliable means to ensure that the most relevant information is prioritized and kept available.

Given the likelihood that initially uncued items would become relevant again upon presentation of the second retrocue, one may question whether participants did, in fact, defocus these items. Based on overall performance (Fig 2A), one may argue that participants held on to all items after the first retrocue, and only dropped the items not cued by the second retrocue. Overall performance would then reflect memory load at the time of retrieval. However, this hypothesis cannot account for the differences in performance for continuously cued (“focused”) and intermittently uncued (“defocused”) items in the Add conditions: Performance for these items should be equal if the first retrocue was ignored, and they should be equally affected by differences in memory load. Instead, performance for continuously cued items was at the same level in all cued conditions, and performance for intermittently uncued items reflected the likelihood with which these items might become relevant again. These findings clearly demonstrate that the information maintained in VWM was weighted according to the probability structure introduced by the cues. How exactly this behaviourally evident sensitivity to a given probability structure is implemented at the representational level in VWM cannot be conclusively determined based on our own findings. In light of evidence for different representational states in VWM that are linked to the attentional status of representations [24–28], we argue that this weighting is implemented by maintenance within and outside the FoA, and that, consequently, initially uncued items were indeed defocused. (Another explanation for the sensitivity to the probability structure could be based on a different interpretation of mean performance. According to this interpretation, mean performance would not be representative of what happened in each trial (i.e., defocusing), but would be a mixture of different processes in different trials (i.e., continued maintenance vs. removal).)

No converging conclusions with regard to this first question we addressed could be drawn from the CDA/SPCN results. Implications of the present findings for the interpretation of the CDA/SPCN shall be discussed in more detail below.

Second, we asked whether the individual efficiency of attentional orienting within VWM was related to the magnitude of the retrocueing benefit. Indeed, stronger N2pc modulations indicating a higher attentional efficiency were associated with larger retrocueing benefits. The finding that individual differences in attentional selection, as indicated by the N2pc, were related to the updating of VWM following retrocues adds to a growing body of literature investigating individual differences in attentional control related to working memory functions. For example, Vogel et al. [62] found differences in the individual efficiency with which only relevant items were selected for encoding, and Fukuda and Vogel [63,64] found that individuals with a high working memory capacity were better at resisting attentional capture by irrelevant distractors. However, as discussed below, the cognitive processes reflected by the N2pc in the present experiment are disputable. Consequently, the relationship between individual N2pc modulations and retrocueing benefits might not be as straightforward as suggested by these findings.

### The CDA/SPCN and the internal focus of attention

The prevailing view of the CDA/SPCN is that it reflects the number of items maintained in VWM. This notion, however, cannot account for the present findings: CDA/SPCN amplitudes did not mirror memory load in the experimental conditions. This was most obvious after the first retrocue, when memory load in the neutral condition was at least as high as in the cued conditions, but CDA/SPCN amplitude was smaller. After the second retrocue, no differences between experimental conditions and, in fact, no overall CDA/SPCN was observed. This may be related to the long trial duration, as the CDA/SPCN has been shown to “naturally” decline over time [42].

The finding of larger amplitudes in the cued conditions than in the neutral condition after the first retrocue could be seen as at variance with those of Kuo et al. [11]. In particular, they observed a stronger CDA/SPCN attenuation after informative retrocues than after neutral retrocues. There is one important difference between our task and the one used by Kuo et al. [11], which could account for the divergent findings. Whereas in their experiments, uncued items were never tested, in our experiment, there was a relatively high likelihood that the initially uncued items would become relevant again. As discussed above, this likelihood might be a crucial factor in determining what happens with uncued information. Thus, the retrocue might have resulted in the removal of uncued information in the experiments by Kuo et al. [11], whereas in our experiment, it might have induced an updating process to differentially weigh and maintain all items. It seems reasonable to assume that such different implications of the cues would yield different patterns of CDA/SPCN amplitudes.

Our findings suggest that the CDA/SPCN is, at least to some degree, associated with the internal FoA. Note that we do not suggest a reinterpretation of the CDA/SPCN as an index of the amount of items in the FoA, but only that there is a link to attentional processes involved in updating VWM according to changes in the task-relevance of specific items, that is, in adjusting the FoA. This idea would account for the pattern of CDA/SPCN amplitudes observed after the memory array and after the first retrocue. After the memory array, the initial FoA was identical in all conditions, which was reflected in equivalent CDA/SPCN amplitudes. After the first retrocue, differential weighting of maintained items was induced in the cued conditions, yielding larger CDA/SPCN amplitudes in the cued compared to the neutral condition.

The conclusions suggested by our present findings are consistent with some previous studies linking the CDA/SPCN to attentional processes. Evidence is provided by neuroimaging studies. The most discussed candidate for the neural source of the CDA/SPCN is the intra-parietal sulcus (IPS). Several studies have observed load-dependent (BOLD) activations reaching a plateau at the mean VWM capacity limit on the group level, which was correlated with VWM capacity on the individual level [65–68]. Load-dependent activations in the IPS have also been observed in tracking tasks varying the attentional load [69,70]. This matches findings on the CDA/SPCN that typically exhibits tracking load-dependent amplitudes [46,53]. Further evidence comes from studies not directly investigating the CDA/SPCN, but the nature of delay activity per se. Two studies using MVPA recorded in event-related fMRI [26] and of electrophysiological activity [24] found evidence for delay activity only for items maintained in the FoA. The authors call into question the long-standing idea that active maintenance is necessary for short-term retention. One does, however, not need to go this far to presume that the CDA/SPCN is related to the internal FoA, given that in most studies establishing the link between CDA/SPCN amplitude and maintained items, the attending and memorizing of items was confounded (e.g., [42,62,71]).

### The N2pc—an electrophysiological marker of selection in VWM?

An N2pc was observed following each retrocue, exhibiting an amplitude modulation that reflected the attentional demands posed by the respective retrocue and that, on the individual level, was related to the magnitude of the benefit for focused items. After the first retrocue, N2pc amplitude was larger in the cued conditions as compared to the neutral condition, in which no attentional updating of VWM was required. After the second retrocue, N2pc amplitudes diverged in the cued conditions compared to after the first retrocue, with larger amplitudes in the Add conditions, in which further updating was necessary. While the N2pc as an index of attention deployment towards targets in extrapersonal space has been extensively studied, only few studies employed this ERP to shed light on mnemonic processes. An N2pc response has been found in studies comparing visual search to search in VWM, with the target being presented before or after a search array [15,72]. While the N2pc for VWM search was temporally and largely topographically equivalent to the N2pc for visual search, it was insensitive to search load. In these tasks, however, only one target had to be selected and the number of distractors, that is, of memory load in VWM search, was varied. In the present study, in contrast, initial memory load was constant and the number of selected memory items was varied. Similar in this respect are multiple object tracking studies asking participants to memorize and track different numbers of objects. Drew and Vogel [46] recorded ERPs during a tracking task, and observed an N2pc that was strongly modulated by the number of target items. This fits nicely to the observation made in the present study, that the larger the number of items to be selected, the larger N2pc amplitude. Importantly, moreover, Drew and Vogel [46] demonstrated that N2pc amplitude reached an asymptote when the mean capacity limit of three to four items [7,8] was exceeded. They also observed that for low-capacity performers, amplitude dropped below the three-item level for set sizes beyond the capacity limit. A similar pattern emerged in the present experiment: N2pc amplitude was larger the more items were to be selected, with the exception of the Add2 condition, which required participants to attend to four items. Here, N2pc amplitude slightly dropped below the level of the Add1 condition (Fig 3). It appears that asking participants to attend to a number of items exceeding their VWM capacity has a disruptive effect on the mechanisms selecting targets in memory, and thus on updating items in VWM according to changed task relevance.

Even though the N2pc responses are consistent with what one would expect for the selection of VWM representations in the present paradigm and fit with previous findings discussed above, it is possible that the N2pc observed in the present study reflected the focusing of attention onto the cue itself. This would have resulted in the same pattern of results. Van Velzen and Eimer [73] showed that centrally presented cues with lateralized components can elicit lateralized ERPs, and concluded that under such conditions, the N2pc reflects the selection of relevant aspects of the cue. Although it should be noted that the cues employed by Van Velzen and Eimer [73] were larger (3.5° vs. 1.1° in the present experiment) and thus extended farther into the visual hemifields, it is possible that this is what was being observed in the present study. According to this alternative scenario, the N2pc responses following the retrocues reflected the selection of the individual corners of the cues, yielding larger N2pc amplitudes in the cued conditions than in the neutral condition after the first retrocue, and even larger amplitudes in the Add conditions than in the Hold condition after the second retrocue. The correlations would correspondingly indicate that individuals who were better at selecting and individuating the corners of the cue were the ones with larger retrocueing benefits. This correlation would presumably still be mediated by better attentional selection in VWM that relies on better attentional processing of the cue. Thus, the N2pc observed here might, in fact, not be a direct electrophysiological correlate of the internal selection of information maintained in VWM, but a correlate of the external selection of the cue, which is a prerequisite for this internal selection. Given that we cannot entirely rule out this alternative scenario, any conclusions drawn on the present N2pc results should be taken with caution.

## Conclusions

The present experiment provides support for the assumption that within VWM, attentional selection is employed to protect those representations which hold particular relevance for our current goals. Defocused representations that may become relevant again are not lost, but can be refocused and recalled. However, defocusing comes at a cost, and memory for the corresponding items is worse than for items that are continuously focused and protected. These findings indicate that maintained information in VWM can be flexibly weighted according to its relevance. The results also provide new insight into the cognitive processes reflected by the CDA/SPCN: Taken together with other findings discussed above, they suggest a linkage between the CDA/SPCN and attentional processes.

## Supporting Information

S1 Data Accuracy. (XLS)

S2 Data Reaction Time. (XLS)

S3 Data Control Experiment (Accuracy). (XLS)

S4 Data Electrophysiological data. Mean amplitudes of ipsilateral and contralateral activity (Sheet 1) and of the difference waves (Sheet 2) for all CDA/SPCN and N2pc time windows of analysis.
(XLS)

S5 Data Correlation data. Accuracy and N2pc and CDA/SPCN amplitudes for the windows of analysis after the retrocues in the Add1 condition relative to baseline (i.e., minus the values in the neutral condition), as well as all of these measures computed separately from even-numbered and odd-numbered trials.
(XLS)

S6 Data Scalp distribution data (normalized). (XLS)

S1 Fig Grand-averaged ERPs. ERPs are shown for the experimental conditions (Hold in red, Add1 in blue, Add2 in green and Neutral in grey) time-locked to the onset of the memory array averaged across parieto-occipital electrodes (PO3/PO4, PO7/PO8). Time windows of stimulus presentations are shaded in grey. For illustration purposes, the waveforms were lowpass filtered (half- amplitude cutoff at 35 Hz, 24 dB/oct).
(TIF)

S2 Fig Grand-averaged contralateral and ipsilateral activity. Contralateral (dashed lines) and ipsilateral (solid lines) activity is shown separately for the four conditions, time-locked to the onset of the memory array and averaged across parieto-occipital electrodes (PO3/PO4, PO7/PO8). Time windows of stimulus presentations are shaded in grey. For illustration purposes, the waveforms were lowpass filtered (half-amplitude cutoff at 35 Hz, 24 dB/oct).
(TIF)

S3 Fig Scalp distribution maps. Scalp distributions of the N2pc and the CDA/SPCN for the time windows of analysis following the first retrocue: 1200–1300 ms for the N2pc and 1450–1650 ms for the CDA/SPCN, time-locked to the onset of the memory array. The left panel shows the scalp distribution of the N2pc on a scale from -2.0 μv to 0 μv, the right panel shows the scalp distribution of the CDA/SPCN on a scale from -0.5 μv to 0 μv. Each panel shows the difference between contralateral and ipsilateral activity averaged across the cued conditions (Hold, Add1 and Add2) and across hemispheres.
(TIF)

S4 Fig Experimental conditions, retrocues and results of the control experiment. **A** In the cued conditions, the first retrocue indicated two positions of previously presented memory items, i.e. the upper or lower quadrant of the respective hemifield. In the neutral condition, the retrocue provided no spatial information. The second retrocue was in the cued conditions either identical to the first one (Hold), additionally marked the adjacent position (Add1) or the whole hemifield (Add2). In the neutral condition, the second retrocue was identical to the first one. **B** Accuracy in percent for each experimental condition (Hold in red, Add1 in blue, Add2 in green, Switch in orange and Neutral in grey). Error bars show the standard errors of the means.
(TIF)

## References

[1] Awh E, Jonides J (2001) Overlapping mechanisms of attention and spatial working memory. Trends in Cognitive Sciences 5: 119–126. 11239812 10.1016/s1364-6613(00)01593-x

[2] Chun MM (2011) Visual working memory as visual attention sustained internally over time. Neuropsychologia 49: 1407–1409. 10.1016/j.neuropsychologia.2011.01.029 21295047

[3] Gazzaley A, Nobre AC (2012) Top-down modulation: bridging selective attention and working memory. Trends in Cognitive Sciences 16: 129–35. 10.1016/j.tics.2011.11.014 22209601 PMC3510782

[4] Kiyonaga A, Egner T (2013) Working memory as internal attention: toward an integrative account of internal and external selection processes. Psychonomic Bulletin & Review 20: 228–242. 23233157 10.3758/s13423-012-0359-y PMC3594067

[5] Mayer JS, Bittner RA, Nikolić D, Bledowski C, Goebel R, Linden DEJ (2007) Common neural substrates for visual working memory and attention. NeuroImage 36: 441–453. 17462914 10.1016/j.neuroimage.2007.03.007

[6] Olivers CNL, Peters J, Houtkamp R, Roelfsema PR (2011) Different states in visual working memory: when it guides attention and when it does not. Trends in Cognitive Sciences 15: 327–34. 10.1016/j.tics.2011.05.004 21665518

[7] Cowan N (2001) The magical number 4 in short-term memory: a reconsideration of mental storage capacity. The Behavioral and Brain Sciences 24: 87–114. 11515286 10.1017/s0140525x01003922

[8] Luck SJ, Vogel EK (1997) The capacity of visual working memory for features and conjunctions. Nature 390: 279–281. 9384378 10.1038/36846

[9] Astle DE, Summerfield J, Griffin IC, Nobre AC (2012) Orienting attention to locations in mental representations. Attention, Perception & Psychophysics 74: 146–162. 10.3758/s13414-011-0218-3 PMC4152722 21972046

[10] Griffin IC, Nobre AC (2003) Orienting attention to locations in internal representations. Journal of Cognitive Neuroscience 15: 1176–1194. 14709235 10.1162/089892903322598139

[11] Kuo B-C, Stokes MG, Nobre AC (2012) Attention modulates maintenance of representations in visual short-term memory. Journal of Cognitive Neuroscience 24: 51–60. 10.1162/jocn_a_00087 21736457 PMC3480577

[12] Lepsien J, Griffin IC, Devlin JT, Nobre AC (2005) Directing spatial attention in mental representations: Interactions between attentional orienting and working-memory load. Neuroimage 26: 733–743. 15955482 10.1016/j.neuroimage.2005.02.026

[13] Lepsien J, Thornton I, Nobre AC (2011) Modulation of working-memory maintenance by directed attention. Neuropsychologia 49: 1569–1577. 10.1016/j.neuropsychologia.2011.03.011 21420421

[14] Astle DE, Scerif G, Kuo B-C, Nobre AC (2009) Spatial selection of features within perceived and remembered objects. Frontiers in Human Neuroscience 3: 1–9. 19434243 10.3389/neuro.09.006.2009 PMC2679200

[15] Dell’Acqua R, Sessa P, Toffanin P, Luria R, Jolicoeur P (2010) Orienting attention to objects in visual short-term memory. Neuropsychologia 48: 419–428. 10.1016/j.neuropsychologia.2009.09.033 19804791

[16] Lepsien J, Nobre AC (2007) Attentional modulation of object representations in working memory. Cerebral Cortex 17: 2072–2083. 17099066 10.1093/cercor/bhl116

[17] Nee DE, Jonides J (2009) Common and distinct neural correlates of perceptual and memorial selection. NeuroImage 45: 963–975. 19280708 10.1016/j.neuroimage.2009.01.005 PMC2775536

[18] Nobre AC, Coull JT, Maquet P, Frith CD, Vandenberghe R, Mesulam MM (2004) Orienting attention to locations in perceptual versus mental representations. Journal of Cognitive Neuroscience 16: 363–373. 15072672 10.1162/089892904322926700

[19] Poch C, Campo P, Barnes GR (2014) Modulation of alpha and gamma oscillations related to retrospectively orienting attention within working memory. European Journal of Neuroscience 40: 2399–2405. 10.1111/ejn.12589 24750388 PMC4215597

[20] Tamber-Rosenau BJ, Esterman M, Chiu Y-C, Yantis S (2011) Cortical mechanisms of cognitive control for shifting attention in vision and working memory. Journal of Cognitive Neuroscience 23: 2905–2919. 10.1162/jocn.2011.21608 21291314 PMC3158824

[21] Cowan N (1993) Activation, attention, and short-term memory. Memory & Cognition 21: 162–167. 8469124 10.3758/bf03202728

[22] Oberauer K (2002) Access to information in working memory: Exploring the focus of attention. Journal of Experimental Psychology: Learning, Memory, and Cognition 28: 411–421. 12018494

[23] Olivers CNL, Eimer M (2011) On the difference between working memory and attentional set. Neuropsychologia 49: 1553–1558. 10.1016/j.neuropsychologia.2010.11.033 21145332

[24] LaRocque JJ, Lewis-Peacock JA, Drysdale AT, Oberauer K, Postle BR (2013) Decoding attended information in short-term memory: an EEG study. Journal of Cognitive Neuroscience 25: 127–142. 10.1162/jocn_a_00305 23198894 PMC3775605

[25] LaRocque JJ, Lewis-Peacock JA, Postle BR (2014) Multiple neural states of representation in short-term memory? It’s a matter of attention. Frontiers in Human Neuroscience 8: 1–14. 24478671 10.3389/fnhum.2014.00005 PMC3899521

[26] Lewis-Peacock JA, Drysdale AT, Oberauer K, Postle BR (2012) Neural evidence for a distinction between short-term memory and the focus of attention. Journal of Cognitive Neuroscience 24: 61–79. 10.1162/jocn_a_00140 21955164 PMC3222712

[27] Nee DE, Jonides J (2013) Neural evidence for a 3-state model of visual short-term memory. NeuroImage 74: 1–11. 10.1016/j.neuroimage.2013.02.019 23435212 PMC3762260

[28] Oberauer K, Hein L (2012) Attention to Information in Working Memory. Current Directions in Psychological Science 21: 164–169.

[29] Rerko L, Oberauer K (2013) Focused, Unfocused, and Defocused Information in Working Memory. Journal of Experimental Psychology: Learning, Memory, and Cognition 39: 1075–1096. 10.1037/a0031172 23421511

[30] Cowan N, Elliott EM, Scott Saults J, Morey CC, Mattox S, Hismjatullina A, Conway ARA (2005) On the capacity of attention: its estimation and its role in working memory and cognitive aptitudes. Cognitive Psychology 51: 42–100. 16039935 10.1016/j.cogpsych.2004.12.001 PMC2673732

[31] Matsukura M, Luck SJ, Vecera SP (2007) Attention effects during visual short-term memory maintenance: protection or prioritization? Perception & Psychophysics 69: 1422–1434. 18078232 10.3758/bf03192957 PMC2150741

[32] Murray AM, Nobre AC, Clark IA, Cravo AM, Stokes MG (2013) Attention restores discrete items to visual short-term memory. Psychological Science 24: 550–556. 10.1177/0956797612457782 23436786 PMC4138001

[33] Landman R, Spekreijse H, Lamme VAF (2003) Large capacity storage of integrated objects before change blindness. Vision Research 43: 149–164. 12536137 10.1016/s0042-6989(02)00402-9

[34] Makovski T, Jiang YV (2007) Distributing versus focusing attention in visual short-term memory. Psychonomic Bulletin & Review 14: 1072–1078. 18229477 10.3758/bf03193093

[35] Sligte IG, Scholte HS, Lamme VAF (2008) Are there multiple visual short-term memory stores? PloS One 3: e1699 10.1371/journal.pone.0001699 18301775 PMC2246033

[36] Sligte IG, Vandenbroucke ARE, Scholte HS, Lamme VAF (2010). Detailed sensory memory, sloppy working memory. Frontiers in Psychology 1: 1–10. 21897823 10.3389/fpsyg.2010.00175 PMC3158431

[37] Pertzov Y, Bays PM, Joseph S, Husain M (2013) Rapid forgetting prevented by retrospective attention cues. Journal of Experimental Psychology. Human Perception and Performance 39: 1224–1231. 10.1037/a0030947 23244045 PMC3793901

[38] Janczyk M, Wienrich C, Kunde W (2008) On the costs of refocusing items in working memory: a matter of inhibition or decay? Memory 16: 374–385. 10.1080/09658210801941742 18432482

[39] Vogel EK, Machizawa MG (2004) Neural activity predicts individual differences in visual working memory capacity. Nature 428: 748–751. 15085132 10.1038/nature02447

[40] Jolicoeur P, Sessa P, Dell’Acqua R, Robitaille N (2006) On the control of visual spatial attention: evidence from human electrophysiology. Psychological Research 70: 414–424. 16184394 10.1007/s00426-005-0008-4

[41] Klaver P, Talsma D, Wijers AA, Heinze H-J, Mulder G (1999) An even-related brain potential correlate of visual short-term memory. NeuroReport 10: 2001–2005. 10424664 10.1097/00001756-199907130-00002

[42] McCollough AW, Machizawa MG, Vogel EK (2007) Electrophysiological measures of maintaining representations in visual working memory. Cortex 43: 77–94. 17334209 10.1016/s0010-9452(08)70447-7

[43] Williams M, Hong SW, Kang M-S, Carlisle NB, Woodman GF (2013) The benefit of forgetting. Psychonomic Bulletin & Review 20: 348–355. 23208769 10.3758/s13423-012-0354-3 PMC3593955

[44] Eimer M (1996) The N2pc component as an indicator of attentional selectivity. Electroencephalography and Clinical Neurophysiology 99: 225–234. 8862112 10.1016/0013-4694(96)95711-9

[45] Luck SJ, Hillyard SA (1994) Electrophysiological correlates of feature analysis during visual search. Psychophysiology 31: 291–308. 8008793 10.1111/j.1469-8986.1994.tb02218.x

[46] Drew TW, Vogel EK (2008) Neural measures of individual differences in selecting and tracking multiple moving objects. The Journal of Neuroscience 28: 4183–4191. 10.1523/JNEUROSCI.0556-08.2008 18417697 PMC2570324

[47] Mazza V, Caramazza A (2011) Temporal brain dynamics of multiple object processing: the flexibility of individuation. PloS One 6: e17453 10.1371/journal.pone.0017453 21387002 PMC3046149

[48] Mazza V, Pagano S, Caramazza A (2013) Multiple Object Individuation and Exact Enumeration. Journal of Cognitive Neuroscience 25: 697–705. 10.1162/jocn_a_00349 23249353

[49] Pagano S, Lombardi L, Mazza V (2014) Brain dynamics of attention and working memory engagement in subitizing. Brain Research 1543: 244–252. 10.1016/j.brainres.2013.11.025 24309139

[50] Pagano S, Mazza V (2012) Individuation of multiple targets during visual enumeration: new insights from electrophysiology. Neuropsychologia 50: 754–761. 10.1016/j.neuropsychologia.2012.01.009 22266261

[51] Woodman GF, Vogel EK (2008) Selective storage and maintenance of an object’s features in visual working memory. Psychonomic Bulletin & Review 15: 223–229. 18605507 10.3758/pbr.15.1.223

[52] Machizawa MG, Goh CCW, Driver J (2012). Human visual short-term memory precision can be varied at will when the number of retained items is low. Psychological Science 23: 554–559. 10.1177/0956797611431988 22527526

[53] Drew TW, Horowitz TS, Wolfe JM, Vogel EK (2012) Neural measures of dynamic changes in attentive tracking load. Journal of Cognitive Neuroscience 24: 440–450. 10.1162/jocn_a_00107 21812558

[54] Ishihara S (2010) Ishihara’s tests for colour deficiency Tokyo, Japan: Kanehara Trading Inc.

[55] Luck SJ (2005) An introduction to the event-related potential technique Cambridge, MA: MIT.

[56] McCarthy G, Wood CC (1985) Scalp distributions of event-related potentials: an ambiguity associated with analysis of variance models. Electroencephalography and Clinical Neurophysiology 62: 203–208. 2581760 10.1016/0168-5597(85)90015-2

[57] Brown W (1910) Some experimental results in the correlation of mental abilities. British Journal of Psychology 3: 296–322.

[58] Spearman C (1910) Correlation calculated from faulty data. British Journal of Psychology 3: 271–295.

[59] Zokaei N, Ning S, Manohar S, Feredoes E, Husain M (2014) Flexibility of representational states in working memory. Frontiers in Human Neuroscience 8: 1–12. 25414654 10.3389/fnhum.2014.00853 PMC4222142

[60] Gunseli E, van Moorselaar D, Meeter M, Olivers CNL (2015) The reliability of retro-cues determines the fate of noncued visual working memory representations. Psychonomic Bulletin & Review 22: 1334–1341. 25563713 10.3758/s13423-014-0796-x

[61] Van Moorselaar D, Olivers CNL, Theeuwes J, Lamme VAF, Sligte IG (2015). Forgotten but not gone: retro-cue costs and benefits in a double-cueing paradigm suggest multiple states in visual short-term memory. Journal of Experimental Psychology: Learning, Memory, and Cognition 41: 1755–1763. 10.1037/xlm0000124 25867613

[62] Vogel EK, McCollough AW, Machizawa MG (2005) Neural measures reveal individual differences in controlling access to working memory. Nature 438: 500–503. 16306992 10.1038/nature04171

[63] Fukuda K, Vogel EK (2009) Human variation in overriding attentional capture. The Journal of Neuroscience 29: 8726–8733. 10.1523/JNEUROSCI.2145-09.2009 19587279 PMC6664881

[64] Fukuda K, Vogel EK (2011) Individual differences in recovery time from attentional capture. Psychological Science 22: 361–368. 10.1177/0956797611398493 21310945 PMC4494671

[65] Robitaille N, Marois R, Todd JJ, Grimault S, Cheyne D, Jolicoeur P (2010) Distinguishing between lateralized and nonlateralized brain activity associated with visual short-term memory: fMRI, MEG, and EEG evidence from the same observers. NeuroImage 53: 1334–1345. 10.1016/j.neuroimage.2010.07.027 20643214

[66] Todd JJ, Marois R (2004) Capacity limit of visual short-term memory in human posterior parietal cortex. Nature 428: 751–754. 15085133 10.1038/nature02466

[67] Todd JJ, Marois R (2005) Posterior parietal cortex activity predicts individual differences in visual short-term memory capacity. Cognitive, Affective & Behavioral Neuroscience 5: 144–155. 10.3758/cabn.5.2.144 16180621

[68] Xu Y, Chun MM (2006) Dissociable neural mechanisms supporting visual short-term memory for objects. Nature 440: 91–95. 16382240 10.1038/nature04262

[69] Culham JC, Cavanagh P, Kanwisher NG (2001) Attention response functions: characterizing brain areas using fMRI activation during parametric variations of attentional load. Neuron 32: 737–745. 11719212 10.1016/s0896-6273(01)00499-8

[70] Jovicich J, Peters RJ, Koch C, Braun J, Chang L, Ernst T (2001) Brain areas specific for attentional load in a motion-tracking task. Journal of Cognitive Neuroscience 13: 1048–1058. 11784443 10.1162/089892901753294347

[71] Ikkai A, McCollough AW, Vogel EK (2010) Contralateral delay activity provides a neural measure of the number of representations in visual working memory. Journal of Neurophysiology 103: 1963–1968. 10.1152/jn.00978.2009 20147415 PMC2853266

[72] Kuo B-C, Rao A, Lepsien J, Nobre AC (2009) Searching for targets within the spatial layout of visual short-term memory. The Journal of Neuroscience 29: 8032–8038. 10.1523/JNEUROSCI.0952-09.2009 19553443 PMC6666054

[73] Van Velzen J, Eimer M (2003) Early posterior ERP components do not reflect the control of attentional shifts toward expected peripheral events. Psychophysiology 40: 827–831. 14696736 10.1111/1469-8986.00083

