# Supplementary figures and images for: The Focus of Attention in Visual Working Memory: Protection of Focused Representations and Its Individual Variation
Source: PLoS One. 2016 Apr 21;11(4):e0154228. doi: 10.1371/journal.pone.0154228 (PMC4839725; doi:10.1371/journal.pone.0154228)

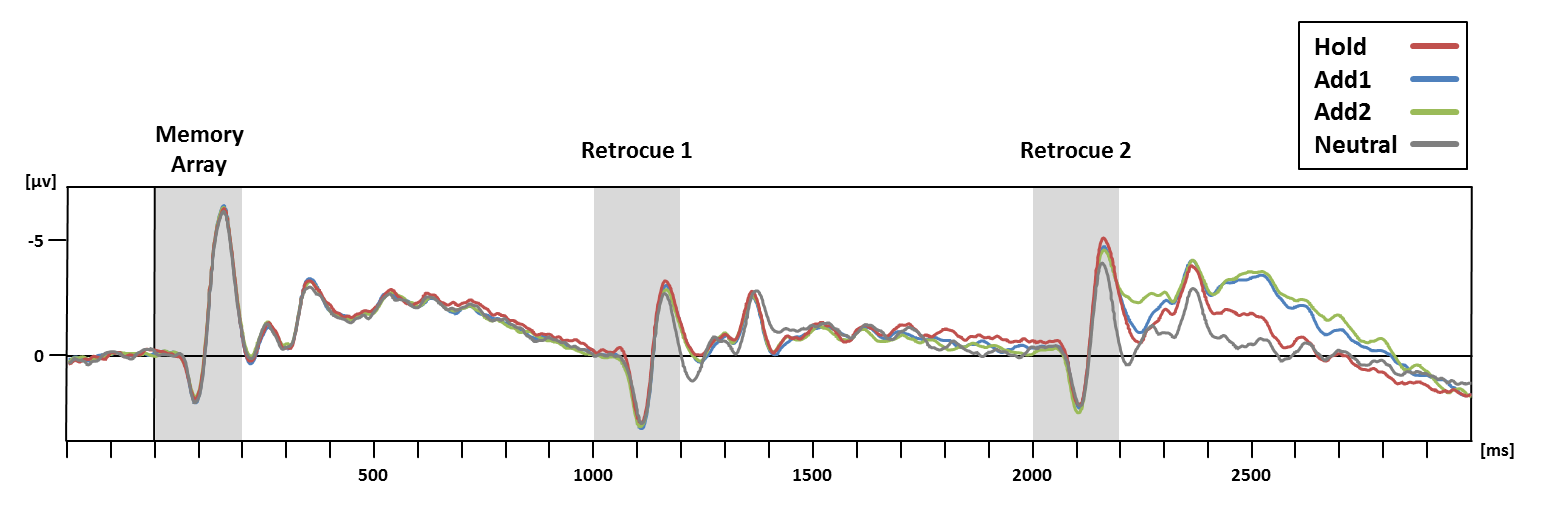

Supplement: S1 Fig — ERPs are shown for the experimental conditions (Hold in red, Add1 in blue, Add2 in green and Neutral in grey) time-locked to the onset of the memory array averaged across parieto-occipital electrodes (PO3/PO4, PO7/PO8). Time windows of stimulus presentations are shaded in grey. For illustration purposes, the waveforms were lowpass filtered (half- amplitude cutoff at 35 Hz, 24 dB/oct). (TIF) [file pone.0154228.s007.tif]

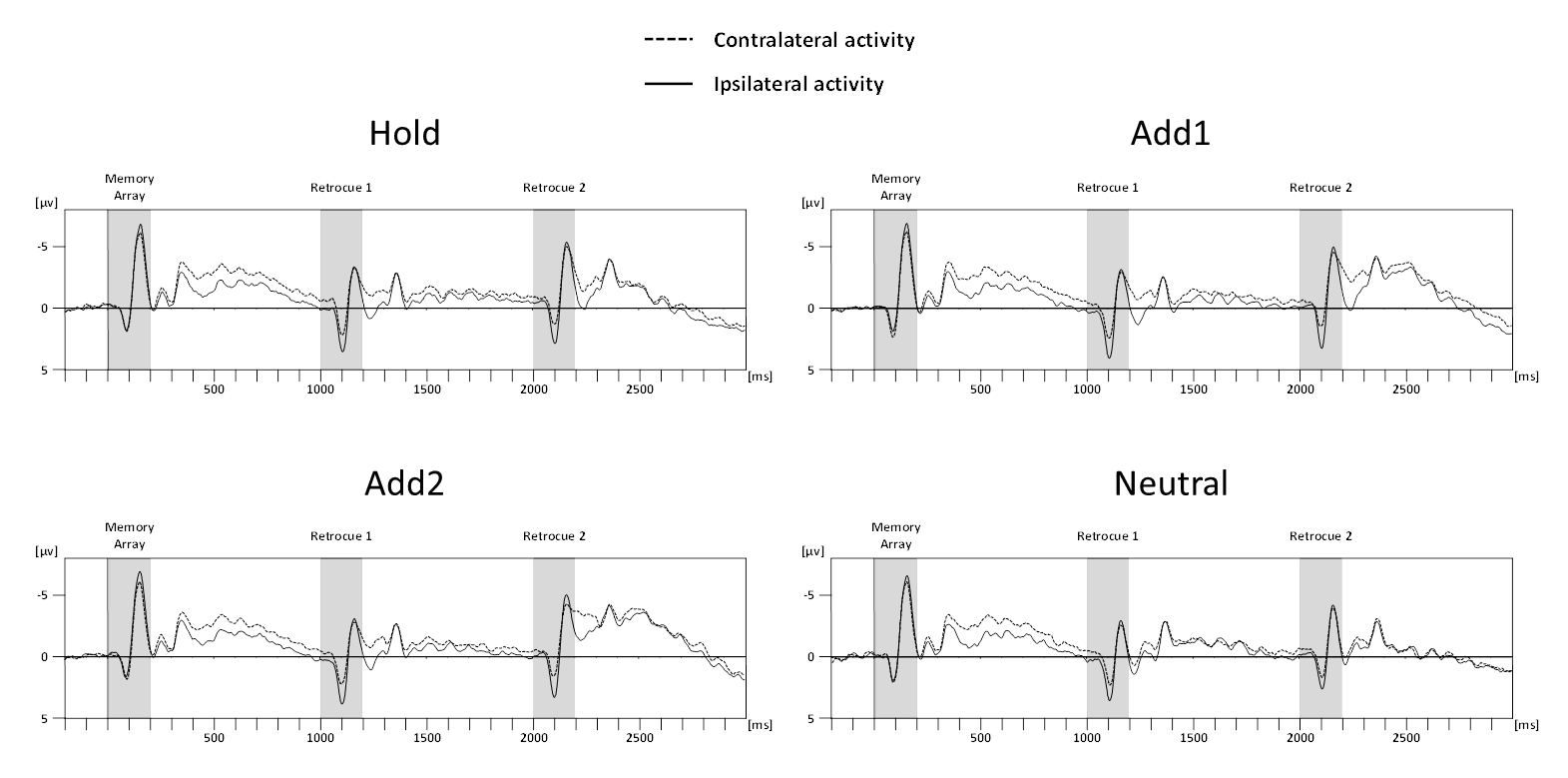

Supplement: S2 Fig — Contralateral (dashed lines) and ipsilateral (solid lines) activity is shown separately for the four conditions, time-locked to the onset of the memory array and averaged across parieto-occipital electrodes (PO3/PO4, PO7/PO8). Time windows of stimulus presentations are shaded in grey. For illustration purposes, the waveforms were lowpass filtered (half-amplitude cutoff at 35 Hz, 24 dB/oct). (TIF) [file pone.0154228.s008.tif]

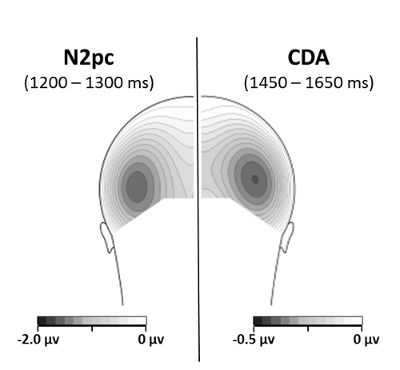

Supplement: S3 Fig — Scalp distributions of the N2pc and the CDA/SPCN for the time windows of analysis following the first retrocue: 1200–1300 ms for the N2pc and 1450–1650 ms for the CDA/SPCN, time-locked to the onset of the memory array. The left panel shows the scalp distribution of the N2pc on a scale from -2.0 μv to 0 μv, the right panel shows the scalp distribution of the CDA/SPCN on a scale from -0.5 μv to 0 μv. Each panel shows the difference between contralateral and ipsilateral activity averaged across the cued conditions (Hold, Add1 and Add2) and across hemispheres. (TIF) [file pone.0154228.s009.tif]

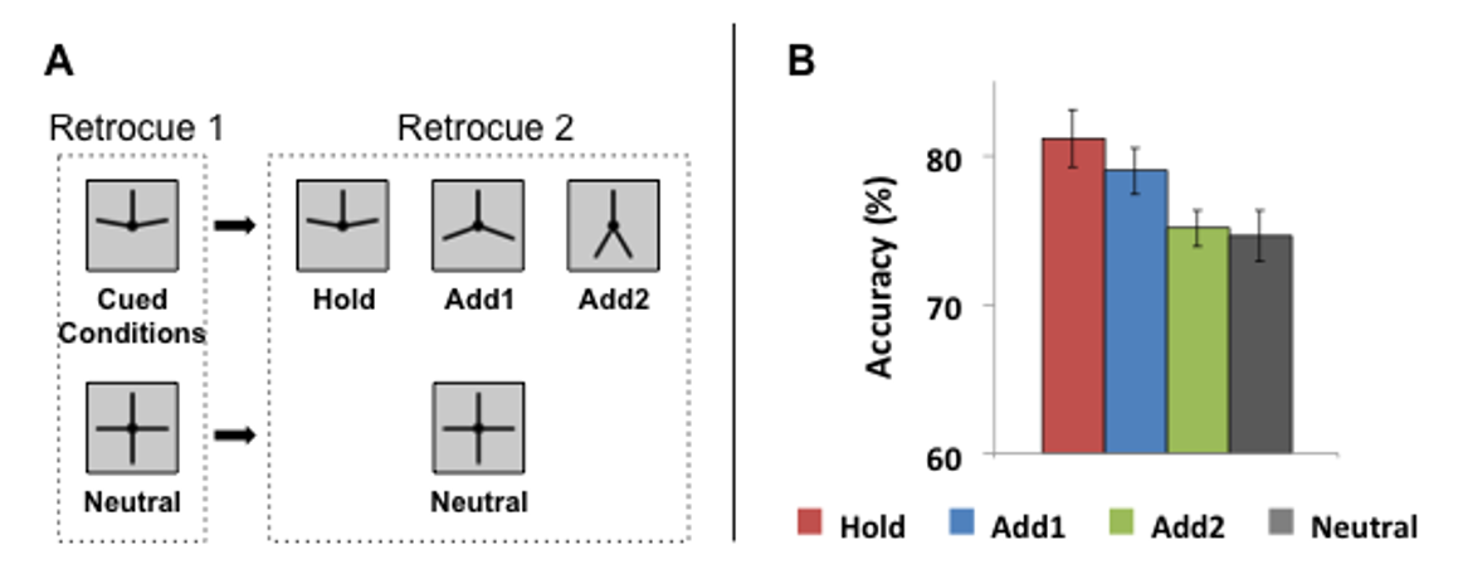

Supplement: S4 Fig — A In the cued conditions, the first retrocue indicated two positions of previously presented memory items, i.e. the upper or lower quadrant of the respective hemifield. In the neutral condition, the retrocue provided no spatial information. The second retrocue was in the cued conditions either identical to the first one (Hold), additionally marked the adjacent position (Add1) or the whole hemifield (Add2). In the neutral condition, the second retrocue was identical to the first one. B Accuracy in percent for each experimental condition (Hold in red, Add1 in blue, Add2 in green, Switch in orange and Neutral in grey). Error bars show the standard errors of the means. (TIF) [file pone.0154228.s010.tif]
